# External validation of the NUn score for predicting anastomotic leakage after oesophageal resection

**DOI:** 10.1038/s41598-017-10084-9

**Published:** 2017-08-29

**Authors:** Matthias Paireder, Gerd Jomrich, Reza Asari, Ivan Kristo, Andreas Gleiss, Matthias Preusser, Sebastian F. Schoppmann

**Affiliations:** 1 Department of Surgery, Upper GI Service, Comprehensive Cancer Center GET-Unit, Medical University of Vienna, Vienna, Austria; 2 Center for Medical Statistics, Informatics, and Intelligent Systems, Medical University of Vienna, Vienna, Austria; 3 Clinical Division of Oncology, Department of Medicine I and Comprehensive Cancer Center, GET-Unit, Medical University of Vienna, Vienna, Austria

## Abstract

Early detection of anastomotic leakage (AL) after oesophageal resection for malignancy is crucial. This retrospective study validates a risk score, predicting AL, which includes C-reactive protein, albumin and white cell count in patients undergoing oesophageal resection between 2003 and 2014. For validation of the NUn score a receiver operating characteristic (ROC) curve is estimated. Area under the ROC curve (AUC) is reported with 95% confidence interval (CI). Among 258 patients (79.5% male) 32 patients showed signs of anastomotic leakage (12.4%). NUn score in our data has a median of 9.3 (range 6.2–17.6). The odds ratio for AL was 1.31 (CI 1.03–1.67; p = 0.028). AUC for AL was 0.59 (CI 0.47–0.72). Using the original cutoff value of 10, the sensitivity was 45.2% an the specificity was 73.8%. This results in a positive predictive value of 19.4% and a negative predictive value of 90.6%. The proportion of variation in AL occurrence, which is explained by the NUn score, was 2.5% (PEV = 0.025). This study provides evidence for an external validation of a simple risk score for AL after oesophageal resection. In this cohort, the NUn score is not useful due to its poor discrimination.

## Introduction

Surgery still remains crucial in the treatment of oesophageal cancer (EC)^1–3^. Due to improvements in the perioperative treatment, patient selection and technical advances, morbidity and mortality-rates could be significantly reduced over the last years. However, especially pulmonary complications and intrathoracic anastomotic leakage (AL) are still considered as the most serious adverse events^4, 5^. Although treatment of AL and its impact on outcome improved over the years, early detection and intervention is vital^6^.

In 2012, Noble and Underwood (NUn) introduced a novel score describing postoperative assessment and detection of ALs^7^. The NUn score applies routinely run systemic inflammatory biomarkers (albumin, C-reactive protein (CRP) and white cell count) and was designed to predict AL prior to clinical manifestation. In a logistic model, the mentioned values lead to a score, which predicted AL, if the value exceeds the number 10 on the fourth postoperative day (POD 4) with 100% sensitivity and 57% specificity.

However, the attempt to validate this score by Findlay and others, analysing a comparable cohort, showed that they were unable to replicate the findings of the original publication^8^. Major limitation of this validation attempt was that laboratory testing did not state CRP values above 156 ml/l. Thus, a crucial variable of the NUn score made a minute replication impossible.

Aim of our study was to attempt validation of the NUn score and screen for other prognostic factors for morbidity and particular for AL after oesophageal resection.

## Results

### Patients

Two hundred fifty-eight patients (79.5% male) were included in this study. Mean age was 62.8 (10.4 standard deviation) years. One hundred sixty-one patients (62.4%) underwent neoadjuvant treatment, of which 145 (56.2%) patients received chemotherapy (CTH) alone and 16 patients (6.2%) were treated with additional radiation therapy. Adenocarcinoma of the oesophagus (AC) was indication for surgery in 176 (68.2%) patients (AEG I: 143 (55.4%), AEG II: 22 (8.53%), AEG III: 11 (4.26%), according to the Siewert classification), whereas 82 (31.8%) patents had ESCC^9^. Twenty-eight patients (10.85%) received adjuvant chemotherapy. Anastomotic leakage was found in 32 patients (12.4%). Median follow-up was 27 (quartiles 12–53.5) months. Three-year survival rate was 48% and 5-year survival rate was 40%. For further demographic and tumor-related details see Table 1.

**Table 1:** Demographics and Tumor-related details.

Variable	All (n = 258)	no. AL (n = 226)	AL (n = 32)	*p*-Value°
Age, years*	62.8 (10.4)	63.1 (10.5)	60.7 (9.4)	0.221^§^
Gender				
Male	205 (79.46)	178 (86.8)	27 (13.2)	0.64
Female	53 (20.5)	48 (90.57)	5 (9.43)	
Tumor location				
Thoracic	82 (31.8)	63 (76.8)	19 (23.17)	0.001
Siewert Type I	143 (55.4)	131 (91.6)	12 (8.4)	
Siewert Type II	22 (4.6)	21 (95.5)	1 (4.6)	
Siewert Type III	11 (4.3)	11 (100)	0	
Tumor histology, No. (%)				
Adenocarcinoma	176 (68.2)	163 (92.6)	13 (7.39)	0.001^$^
Squamous cell carcinoma	82 (31.8)	63 (76.8)	19 (23.17)	
Neoadjuvant treatment				
Chemo-therapy	161 (62.4)	140 (87.0)	21 (13.0)	0.846
Radiation-therapy	16 (6.2)	14 (87.5)	2 (12.5)	1
Adjuvant treatment				
Chemotherapy	28 (10.85)	24 (85.7)	4 (14.3)	0.761
Tumor Grading				
Well differentiated (G1)	11 (4.3)	7 (63.6)	4 (36.4)	0.196
Moderately differentiated (G2)	109 (42.3)	95 (87.2)	14 (12.8)	
Poorly differentiated (G3)	110 (42.6)	99 (90.0)	11 (10.0)	
Undifferentiated (G4)	25 (9.7)	22 (88.0)	3 (12.0)	
Gx	3 (1.2)	3 (100)	0	
Pathologic tumor stage				
T0	14 (5.4)	11 (78.6)	3 (21.4)	0.213
T1	65 (25.2)	54 (83.08)	11 (16.9)	
T2	46 (17.8)	41 (89.1)	5 (10.9)	
T3	112 (43.4)	102 (91.1)	10 (8.9)	
T4	12 (4.7)	9 (75.0)	3 (25.0)	
Tx	9 (3.5)	9 (100)	0	
Pathologic nodal stage				
N0	127 (49.2)	109 (85.8)	18 (14.17)	0.426
N1	105 (40.7)	93 (88.6)	12 (11.4)	
N2	10 (3.9)	8 (80.0)	2 (520.0)	
N3	15 (5.8)	15 (100)	0	
Nx	1 (0.4)	1 (100)	0	
Surgical margin status				
Clear	240 (93.1)	209 (87.1)	31 (12.9)	0.708
Microscopically involved (R1)	18 (7.0)	17 (94.4)	1 (5.6)	

Values in parentheses are percentages unless indicated otherwise; *Values are mean (standard deviation). AL, anastomotic leakage.

°Fisher’s exact test, ^§^Logistic regression model. ^$^Remains significant after correction for multiple testing using Bonferroni-Holm correction.

### Operation

One hundred sixty-seven (64.7%) patients underwent open oesophagectomy (OE), whereas minimal invasive oesophagectomy (MIE) was performed in 91 (35.2%). This also includes thoracoscopic resection in 29 patients (10%). Abdomino-right-thoracic esophagectomy (Ivor Lewis procedure) was done in 199 (77.1%) patients. The other resections were 34 (13.2%) transhiatal oesophagectomies, 25 (9.6%) transhiatal extended gastrectomies. For further details please see Table 2.

**Table 2:** Procedure Details.

Procedure Type	no AL (n = 226)	AL (n = 32)	*p*-Value°
Abdomino-thoracic (Ivor Lewis)	172 (86.4)	27 (13.6)	0.097
Transhiatal extended gastrectomy	25 (100.0)	0	
Transmediastinal oesophagectomy	29 (85.3)	5 (14.7)	

Values in parentheses are percentages within procedure type, °Fisher’s exact test.

### Anastomotic leakage

Age and gender distributions were similar between patients who did and did not develop AL. Surgical approach as well as length of operation did not significantly influence the AL-rate (*p* = 0.401 and *p* = 0.499). A logistic regression model shows that each additional hour of operation time increases the odds of AL on average by 10% (*p* = 0.499).

Looking at tumor-related details, patients with ESCC were more likely to develop an AL than patients with AC (*p* = 0.001). With respect to other pathological findings, there were no statistically significant differences.

Median time to diagnosis of AL after surgery was 9 days (quartiles 5–10, range 1–23). Thirty (93.8%) patients showed clinical signs of AL, whereas two patients (6.3%) had asymptomatic AL diagnosed with routine contrast swallow. Beside clinical assessment in 11 (34.4%) patients, 12 (37.5%) patients were evaluated with contrast swallow and 9 (28.1%) patients with computed tomography. Sixteen patients (50%) were treated endoscopically (stent implantation), while 7 patients (21.2%) needed re-thoracotomy and closure of the defect. In two patients (6.3%) closure of the defects was not possible due to severe contamination and case of sepsis and the anastomosis was taken down. Seven patients (21.9%) with AL were treated conservatively (nil per os, intravenous antimicrobials and prolonged retaining of drainage).

All patients were extubated after primary surgery within 24 hours. Indication for re-intubation was pneumonia, sceptical condition, recurrent nerve palsy or AL. As expected, patients with AL needed significantly longer duration of ventilation (total days after re-intubation) as well as ICU and hospital stay (*p* < 0.001). Perioperative details are displayed at Table 3.

**Table 3:** Perioperative Details.

Variable	All Total (n = 293)	no AL (n = 259)	AL (n = 34)	*P*-value
Length of operation (minutes)*	330 (300–400)	333 (300–400)	331 (305–418)	0.499°
Duration of ventilation (days)*	0 (0–1)	0 (0–0)	1 (1–6)	<0.001°
ICU stay (days)*	4 (2–7)	0 (0–1)	10 (4–19)	<0.001°
Hospital stay (days)*	14 (11–22)	13 (11–18)	31 (25–42)	<0.001°
Surgical approach**				
Open access	167 (64.7)	146 (87.4)	21 (12.6)	0.401^§^
Minimally invasive	71 (27.5)	64 (90.4)	7 (9.9)	
Laparoscopic assisted	20 (7.7)	16 (80.0)	4 (20.0)	

Values in parentheses are percentages (within total sample; **Within variable category) unless indicated otherwise; *Values are median (quartiles); ICU, intensive care unit; AL, anastomotic leakage; °Logistic regression (applied to log-transformed variable in case of duration of ventilation, ICU stay and hospital stay). ^§^Fisher’s exact test.

### NUn Score

**Figure 1:**
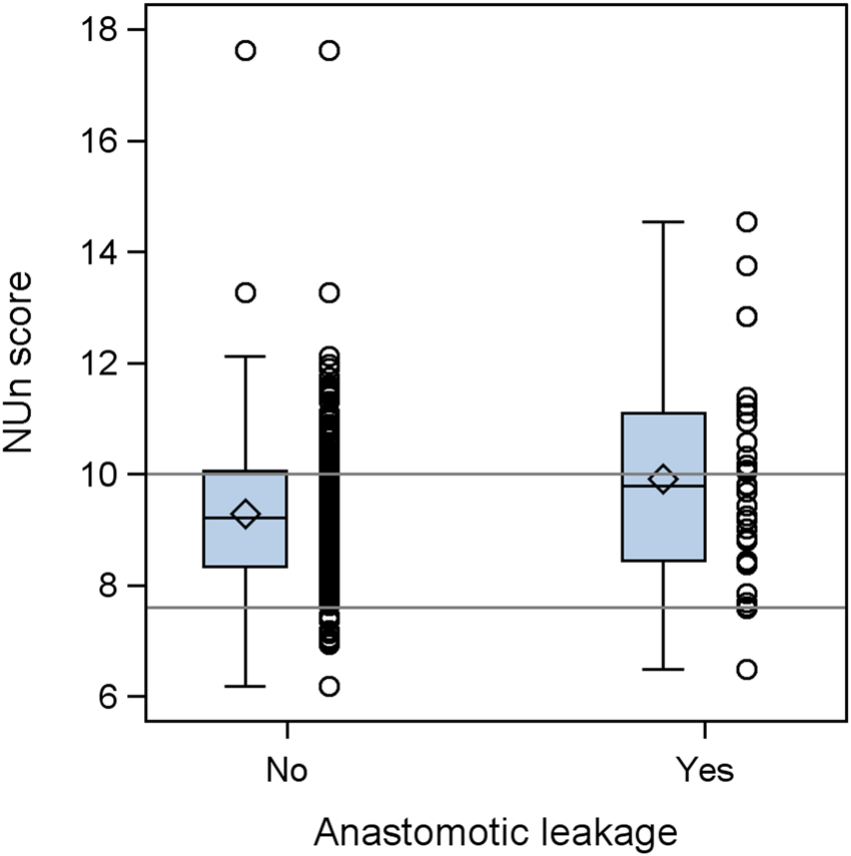
Boxplot and dot plot of NUn score values in patients without and with anastomotic leakage. Horizontal lines at cutoff values 7.6 and 10.0.

**Figure 2:**
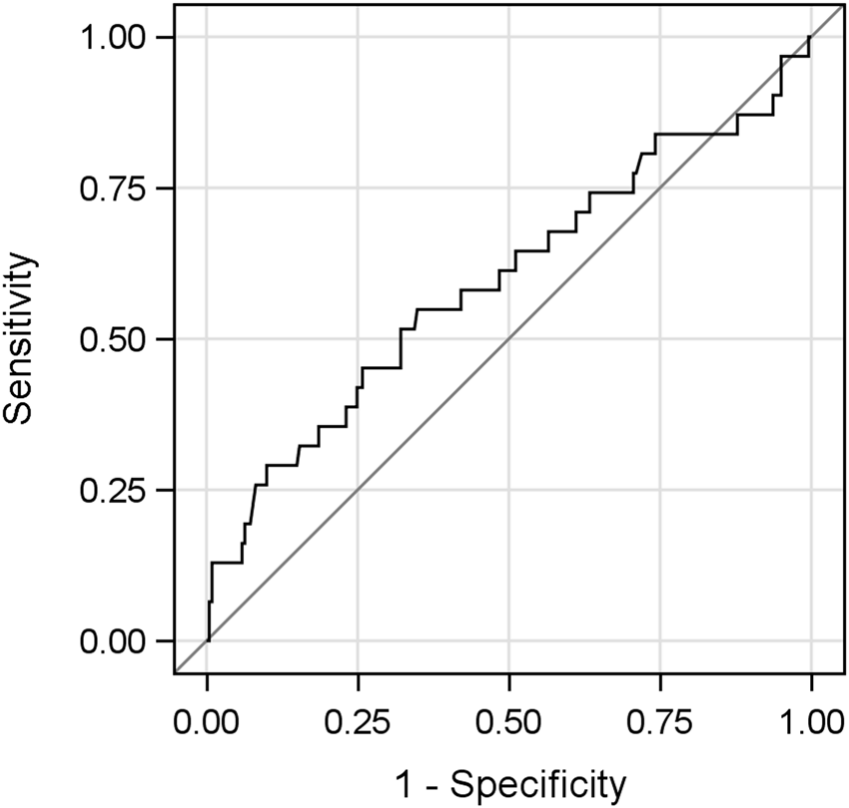
Receiver Operating Characteristic (ROC) curve for NUn score with respect to anastomotic leakage. Area under the ROC curve: 0.59 (95% CI: 0.47–0.72). Diagonal indicates random prediction (coin toss).

The median Nun score observed in our data is 9.3 (quartiles 8.4–10.1, range 6.2–17.6). Box plot and dot plot of NUn score values in patients without and with AL are displayed in Fig. 1. The odds for AL increase on average by 31% with every point on the NUn score (OR of 1.31 (CI 1.03–1.67), p = 0.028). The AUC for AL was 0.59 (CI 0.47–0.72). The Receiver Operating Characteristic (ROC) curve is shown in Fig. 2.

With the original NUn score cutoff value of 10, sensitivity was 45.2% (CI 27.6–62.7) and specificity was 73.8% (CI 68.0–79.6). For the observed prevalence of AL, this results in a positive predictive value of 19.4% and a negative predictive value of 90.6%.

Using a cutoff value of 7.6 as described by Findlay and others in the first validation attempt, the sensitivity increased to 93.6% (CI 84.9–100.0) and the specificity decreased to 5.0% (CI 2.1–7.8) with a positive predictive value of 12.1% and a negative predictive value of 84.6% for the observed AL prevalence.

The proportion variation in AL occurrence, which is explained by the NUn score, amounts to only 2.5% (PEV = 0.025).

**Figure 3:**
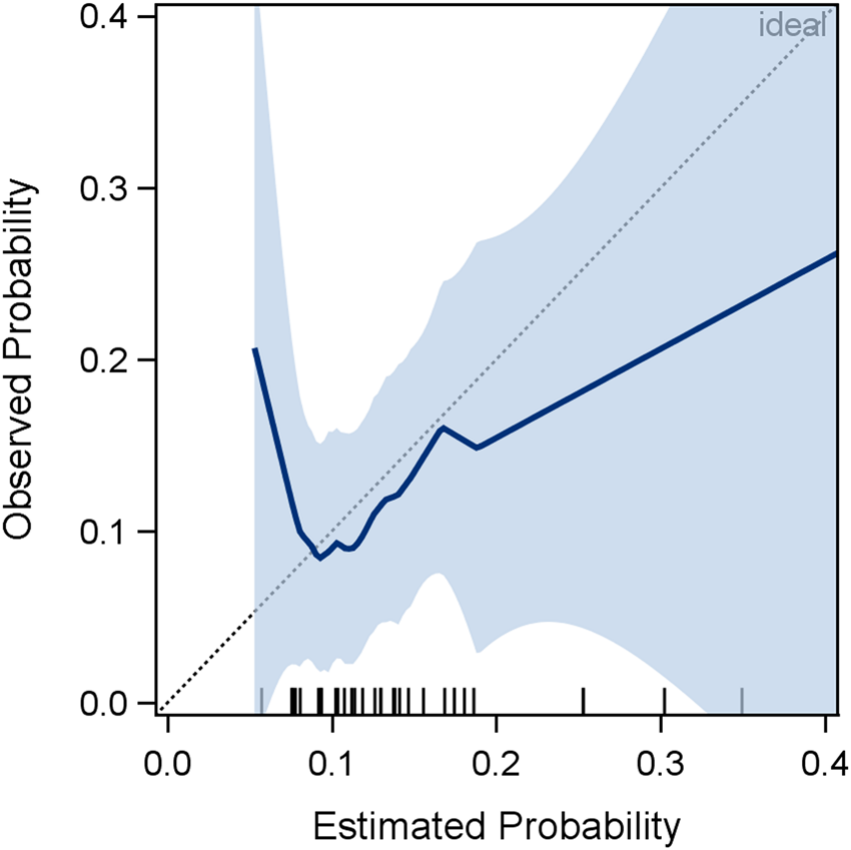
Calibration curve: LOESS smoothed observed probabilities (with 95% confidence band) vs. estimated probabilities for anastomotic leakage. Fringes on horizontal axis indicate estimated probabilities for patients with anastomotic leakage events. Diagonal indicates ideal calibration.

For the purpose of an additional validation of the NUn score a calibration curve was used. Comparing the AL rate in our cohort with the probability of the AL rate predicted with the NUn score, the estimated probability was useful in the range of 0.07 and 0.17. This corresponds to a NUn score between 7.6 and 10.9. The calibration curve is shown in the Fig. 3.

In a decision curve analysis, we assumed that with a reported individual AL risk of 10% or more a patient would agree to preventive measures^10^. This corresponds to considering the harms associated with a missed AL to be nine times greater than the harms associated with unnecessary preventive measures^10^. Based on this assumption there is a small additional net benefit when basing a decision for preventive measures on the NUn score compared to considering no patient at all for preventive measures. If preventive measures are an individual option with an AL risk below 10%, the strategy of using the NUn score is equal to considering every patient.

## Discussion

This study provides evidence for an external validation of a simple score for risk assessment for anastomotic leakage after oesophageal resection. In the herewith presented patient cohort, the NUn score predicted AL significantly. With each point on the NUn score the odds for AL are increased on average by 31%. In a reasonable range of Nun-score values AL probabilities predicted by the Nun-score matched those observed in the sample. Nevertheless, looking at the AUC curve the model is only marginal significantly different from a coin toss. Similarly, the proportion of variation in AL occurrence that is explained by the Nun score is very low (2.5%). This demonstrates that individual prediction based on the Nun-score is poor.

After the negative first external validation by Findlay and others, it was necessary to take their statistical approach into account. Therefore a second calculation was done at their chosen cutoff value at 7.6. Even though the presented results are in good correlation with their findings, this adaption of the cutoff value leads to a significant reduction of the sensitivity and specificity of the NUn score with consequent limitation of clinical usefulness.

Due to high mortality rates in patients with AL and still missing clear treatment algorithms, early detection of AL seems to be mandatory. Therefore a useful score should preferably detect patients before there is clinical evidence.

A recent published study reviewed a large number of existing risk models for outcome after oesophagogastric surgery^11^. They concluded that all existing models, including prediction models for 30-day and 90-day mortality, showed moderate discrimination. Scanning for models predicting AL, they identified seven published risk models^7, 12–17^.

Among the mentioned different risk models for AL, the NUn score is the only model, which evaluates postoperative acute inflammation biomarkers and stated an AUC for presenting its predictive ability. Yet, it was not possible to replicate the initial value of 0.801 and 0.879 respectively.

The hereby presented external validation model uses a patient cohort comparable in size and characteristics, but different to the first validation attempt, there is no threshold when measuring CRP values. Although minimizing this limitation of Findlay *et al*. we could not demonstrate a feasible utility of this risk score.

The utility of a postoperative risk score as such, is as an adjunct to high-quality clinical care. The NUn score as originally described had high sensitivity at the expense of modest specificity meaning that it would never be used alone in clinical decision-making. Rather the NUn score is used to reassure the clinical team that those patients who are making good progress can continue to be fast-tracked. The peri-operative pathway at our institution was adapted to standardized protocols described by Cerfolio *et al*. in 2004^18^. In short, this protocol includes on operation day: epidural catheter, chest anastomosis, prophylactic steroids, antibiotics, fluid restriction, immediate extubation, ICU: continuous positive airway pressure (CPAP) on POD 1: dye swallow, oral fluids, initiation of mobilization, on POD 2: remove left (if <250 ml) and right anterior chest tube, physiotherapy, on POD 3: leaving ICU, remove epidural, start soft diet, on POD 4: contrast swallow, increase soft diet, on POD 5: remove chest tube and central line, complete semisolid diet, on POD 6: dietary instructions, complete mobilization, instructions of subcutaneous injection and between POD 7–12 discharge with instructions, outpatient visit 2–3 weeks after^19^.

Scanning literature there is still need for a postoperative risk model to predict AL at an early stage. Several preoperative risk factors and models were presented to identify a high-risk group, but a postoperative individual model would add a useful instrument to our toolbox. In this cohort, the NUn score is not useful, because it provides poor discrimination.

## Methods

This study is a retrospective analysis of all consecutive patients who underwent oesophageal resection for malignancy of the oesophagus at the Department of Surgery, Medical University of Vienna between 2003 and 2014.

Clinical data was obtained from a prospective institutional database. Laboratory values, from daily routine blood draw, were extracted of the hospital information system. The institutional review board approved this study. Methods were carried out in accordance with relevant guidelines and regulations. Individual informed consent was not acquired, due to study design and national regulations.

Abdominothoracic oesophageal resection was performed in patients with oesophageal squamous cell cancer (ESCC) or adenocarcinoma of the oesophageal-gastric junction (AEG) I and II, transhiatal extended gastrectomy was performed in patients with AEG II and III tumors^9^. Patients who underwent distal oesophagectomy (Merendino procedure) or cervical resection were excluded from this analysis for the benefit of a more homogenous cohort.

All cases were discussed in the interdisciplinary tumor board meeting. Staging included computed tomography scan and positron emission tomography only in special indication. Staging laparoscopy was exclusively performed in AEG III tumors.

Follow up was performed on a 3-month base the first two years and every six months until year 5 after surgery.

### NUn score

NUn score was calculated via the published formula (NUn score = 11.3894 + (0.005 × CRP) + (WCC × 0.186) − (0,174 × albumin). CRP (normal range <0.5 mg/dl; measurement uncertainty: 2.7%) and albumin (normal range: 35–52 g/l; measurement uncertainty: 1.8%) were analysed with the cobas modular analyser series (Roche Diagnostics, Switzerland). White cell count (WCC) (normal range: 4–10 G/l) was analysed with the Sysmex XE-2100 hematological analyser. CRP values were transformed to mg/l for calculation of the Nun score. Unlike other centres there was no CRP threshold for high values. Laboratory values were used from POD 4 as described by Noble *et al*.

### Statistical Analysis

Age is described as mean and standard deviation (SD). Other continuous variables are described as medians and quartiles due to non-normal distributions. Categorical variables are described as counts and percentages. The potential impact of categorical parameters on AL occurrence is exploratively tested using Fisher’s exact tests due to small expected cell counts. The potential impact of continuous variables, including the NUn score, on AL occurrence is investigated using simple logistic regression models.

For the NUn score a receiver operating characteristic (ROC) curve is estimated and the area under the ROC curve (AUC) reported with 95% confidence interval. For previously reported cut-off values of the Nun score sensitivity and specificity are reported with 95% confidence intervals. Positive and negative predicted values are calculated based on the AL prevalence observed in the present data.

3- and 5-year survival probabilities are estimated using the Kaplan-Meier estimator. Median follow-up time is estimated using the reverse Kaplan-Meier method^20^. The proportion of explained variation is calculated using the method of Schemper-Henderson^21^. A calibration curve is produced which contrasts AL probabilities observed in the data with those estimated from the logistic regression model. Observed probabilities are smoothed by LOESS.

The reported p-values are the results of two-sided tests. P-values ≤ 0.05 were considered to be statistically significant. For demographic and tumor-related variables statistical significance after multiplicity correction by the method of Bonferroni-Holm is indicated. All computations were carried out using SAS software Version 9.4 (SAS Institute Inc., Cary, NC, USA, 2012).

The datasets analysed during the current study are available from the corresponding author on reasonable request.
